# T Lymphocytes from Chagasic Patients Are Activated but Lack Proliferative Capacity and Down-Regulate CD28 and CD3ζ

**DOI:** 10.1371/journal.pntd.0002038

**Published:** 2013-01-31

**Authors:** Nicolás A. Giraldo, Natalia I. Bolaños, Adriana Cuellar, Nubia Roa, Zulma Cucunubá, Fernando Rosas, Víctor Velasco, Concepción J. Puerta, John M. González

**Affiliations:** 1 Grupo de Ciencias Básicas Médicas, Facultad de Medicina, Universidad de los Andes, Bogotá, Colombia; 2 Grupo de Inmunobiología y Biología Celular, Facultad de Ciencias, Pontificia Universidad Javeriana, Bogotá, Colombia; 3 Grupo de Trasplante, Facultad de Medicina, Pontificia Universidad Javeriana y Hospital Universitario San Ignacio, Bogotá, Colombia; 4 Grupo de Parasitología, Instituto Nacional de Salud, Bogotá, Colombia; 5 Fundación Clínica Abood Shaio, Bogotá, Colombia; 6 Laboratorio de Parasitología Molecular, Departamento de Microbiología, Facultad de Ciencias, Pontificia Universidad Javeriana, Bogotá, Colombia; Federal University of São Paulo, Brazil

## Abstract

**Background:**

Chronic persistent infections have been associated with T lymphocytes functional impairment. The aim of this study was to compare the activation status, the proliferative potential and the expression of CD28 and CD3ζ chain on T lymphocytes between chronic chagasic patients and uninfected controls.

**Methodology/Principal Findings:**

Forty-two chronic chagasic patients, 28 healthy individuals and 32 non-chagasic cardiomyopathy donors were included. Peripheral blood was marked for CD3, CD4, CD8, HLA-DR, CD28, CD38 and intracellular CD3ζ. Peripheral blood mononuclear cells were stained with carboxyfluorescein diacetate succinimidylester and incubated with *T. cruzi* lysate or phytohemagglutinin for five days. Cells from 3 healthy controls were incubated with *T. cruzi* trypomastigotes separated with transwells; and the expression of CD3ζ chain and proliferation index was determined. Heart-infiltrating cells from two chronic chagasic patients were tested for the aforementioned cellular markers. Chagasic patients displayed higher frequencies of CD4+/HLA-DR+/CD38+ (8.1%±6.1) and CD8+/HLA-DR+/CD38+ (19.8±8.9) T cells in comparison with healthy (1.6±1.0; 10.6±8.0) and non-chagasic cardiomyopathy donors (2.9±2.9; 5.8±6.8). Furthermore, the percentage of CD4+ activated T cells was higher in chagasic patients with cardiac involvement. CD8+ T cells proliferation index in chagasic donors (1.7±0.3) was lower when compared with healthy (2.3±0.3) and non-chagasic cardiomyopathy individuals (3.1±1.1). The frequencies of CD4+/CD28+ and CD8+/CD28+ T cells, as well as the CD3ζ^bright^/CD3ζ^dim^% ratios in CD4+ and CD8+ were lower in chagasic patients when compared with both control groups. The CD3ζ^bright^/CD3ζ^dim^% ratio and proliferative indexes for CD4+ and CD8+ T lymphocytes decreased gradually in those cells cultivated with parasites and displayed lower values than those incubated with medium alone. Finally, heart-infiltrating T cells from two *T. cruzi* infected patients also expressed activation markers and down-regulate CD28 and CD3ζ.

**Conclusions:**

CD8+ T lymphocytes from chagasic donors displayed reduced proliferative capacity, which might be associated with CD3ζ down-regulation and diminished CD28 expression on CD4 T cells.

## Author Summary

In Chagas disease, caused by *Trypanosoma cruzi*, there is an acute onset from which most of the individuals recover if a competent immune response develops. Patients can remain asymptomatic (indeterminate) for many years, but near 30% can develop specific organ damage, mainly the heart. The pathogenesis of chronic Chagas cardiomyopathy is associated with parasite persistence, inadequate immune response and autoimmunity. Here we explored the bulk T cells response of chronic chagasic patients with and without cardiomyopathy, uninfected controls and patients with non-chagasic cardiomyopathy. T cells from chagasic donors expressed higher levels of activation markers, and their co-expression on CD4+ T cells distinguished those donors with or without cardiac damage. Mitogen-induced proliferation is highly impaired in the CD8+ T cells compartment, and interestingly activated CD8+ T cells are negatively correlated with the CD4+/CD28− population. The expression of the CD3ζ chain and CD28 was also diminished in chagasic patients. Furthermore, *in vitro* studies with blood mononuclear cells from uninfected donors showed that the indirect contact with live parasite decreased expression of CD3ζ chain and altered the T cell mitogen-induced proliferation. These results suggest a global impairment of the peripheral T cells response, which is probably associated with parasite persistence.

## Introduction

Upon first contact with an infectious agent, antigen-specific T cells proliferate and rapidly expand their number in order to control or eliminate the microorganism [1]. After effective elimination of the pathogen, this antigen-driven cellular expansion is followed by an aptoptosis-mediated contraction. Successful identification of newly generated effector T cells has been described in several infectious diseases through the co-expression of surface activation markers, CD38 and HLA-DR [2]–[5]. Consistently, only a fraction of these activated CD38+/HLA-DR+ T cells can be detected after the acute infection has been removed [2], [4].

However, in some chronic infections, activated CD38+/HLA-DR+ T cells can be persistently expanded [6], [7] and to some extent correlate with disease progression [8], [9]. Simultaneously with this phenomenon, numerous cellular effector functions including cytokine production, cytotoxic potential and proliferative capacity becomes impaired, in a process termed lymphocyte exhaustion [10], [11]. Previously reported by our group, we show that chronic chagasic patients display higher percentages of CD4+/CD8+ (double-positive) peripheral T cells co-expressing CD38 and HLA-DR when compared with uninfected controls; in addition, the patients with severe cardiomyopathy produced less IFN-γ than those with non-cardiac involvement [12]. The role of the activated subpopulations of T cells in Chagas disease control or pathogenesis requires additional research.

Chagas disease is a chronic parasitic infection caused by the hemoflagellated protozoan *Trypanosoma cruzi*. Clinically, it is possible to distinguish an acute and a chronic phase. After recovery from acute infection most of the individuals remain asymptomatic for several years and nearly 30% of them gradually develop tissue damage including chagasic cardiomyopathy or gastrointestinal involvement [13]. Experimental evidence suggests that the parasite's persistence and the related dysfunctional immune response may contribute to chronic tissue damage [12], [13].

It is claimed that *T. cruzi* persistence can disrupt the normal activation pathways of T lymphocytes and simultaneously induce their exhaustion. For example, patients with severe cardiac involvement had increased percentages of peripheral CD8+ memory T cells with terminal differentiated phenotype (CD8+/CD27−/CD28−) [14] and decreased capacity to produce a *T. cruzi*-specific IFN-γ response than those with mild compromise [12], [15]–[17]. Similarly, severe clinical status in Chagas has been associated with higher expression of inhibitory markers (CTLA-4) on CD8+ T cells [17], CD57 expression and spontaneous apoptosis on CD4+ T cells [18]. Data on T cell activation in chronic Chagas disease is diverse and the evidence can be categorized as follows: a) some studies showed that HLA-DR+ expression on CD3+ [19] or CD4+ [20] cells augments in *T. cruzi* infected patients, but does not vary with disease severity; b) other authors did not found differences between chronically infected patients and controls, whether adults [21], [22] or children were being evaluated [23]; and finally c) other found differences when patients were classified according to disease stage or severity. Specifically, the reports suggest that the percentage of CD4+/HLA-DR+ T cells decreases in the “early-chronic” chagasic children [24], while the CD8+/HLA-DR+ increases in patients with severe cardiac involvement [20], [24].

In other chronic infectious models, similar cellular features as those described above have been related to changes in the expression of molecules involved in T cell activation and downstream signaling. Notably, CD28 and CD3ζ chain down-regulation has been described in several antigen-persistent infectious models and related with diminished proliferation and reduced IFN-γ production on T cells [25], [26]. Therefore, the aim of this study was to compare the activation status, through the co-expression of CD38 and HLA-DR, the proliferative potential and the expression of CD28 and CD3ζ chain on T lymphocytes between chronic chagasic patients and uninfected controls.

## Methods

### Ethics statement

Research protocols and informed consents were approved by the Ethical Committees of the Universidad de los Andes (039-2009), Pontificia Universidad Javeriana (01-2010), Hospital Universitario San Ignacio (77-2011) and the Fundación Abood Shaio (134-2010), Bogotá, Colombia, following the national regulations and the Declaration of Helsinki. All the included subjects provided written informed consent.

### Human donors

One hundred and two volunteers were enrolled in this study and categorized in three groups. The first group included 42 patients with chronic Chagas disease, diagnosed by positive results in both immunofluorescence indirect assay (IFI) and ELISA test. Twenty-seven females and 15 males of ages ranging from 36 to 72 years (X = 52.1, SD±9.9) were recruited at the Fundación Abood Shaio, Hospital Universitario San Ignacio and Instituto Nacional de Salud (Bogotá, Colombia). Patients were classified according to the American College of Cardiology/American Heart Association staging [27] as follows: Ten individuals as A [normal electrocardiogram (ECG) and echocardiogram (ECHO) findings, and New York heart association functional classification (NYHA) I], 11 as B (abnormal ECG findings, normal ECHO and NYHA I), 12 as C (abnormal ECG findings, increased heart size, decreased LVEF according ECHO, and NYHA II or III) and 9 as D (same as class C, but NYHA IV). For statistical analysis, chagasic patients were further divided in two: those with non-structural damage (groups A and B, named non-CARD) and those with structural damage (groups C and D, named CARD). The second group included 28 healthy donors, 20 females and 8 males with ages ranging from 34 to 68 years old (49.7±8.5), with negative IFI and ELISA test results. The third group included 32 seronegative donors, 17 females and 15 males with ages ranging from 28 to 86 years old (59.8±11.4), with non-infectious cardiomyopathy. In this group, cardiomyopathy etiology was: ischemic (n = 14), hypertensive (n = 7), dilated idiopathic (n = 6) and valvular (n = 5). This last group was recruited at the Department of Cardiology, Hospital Universitario San Ignacio, Bogotá, Colombia. The main characteristics of the three groups, denoted as non-CARD and CARD chagasic patients (CP), healthy controls (HC) and non-chagasic cardiomyopathy (NCC) respectively, are shown in Table 1.

**Table 1:** Demographic and clinical characteristics of analyzed individuals.

	Non-CARD Chagasic Patients	CARD Chagasic Patients	Healthy Controls	Non-chagasic Cardiomyopathy	*P* value
Number of individuals	21	21	28	32	-
Median age (range)	49.1 (37–65)	55.4 (40–72)	49.7 (34–68)	59.1 (28–86)	0.46
Male sex (%)	5 (23.8%)	10 (47.6%)	8 (29%)	15 (47%)	0.11
	Clinical assessment				
ACC/AHAS classification					
A No. (%)	10 (47.6%)	-	-	2 (6%)	-
B No. (%)	11 (52.4%)	-	-	4 (12%)	-
C No. (%)	-	12 (57.1%)	-	19 (45%)	-
D No. (%)	-	9 (28.6%)	-	7 (22%)	-
Mean LVEF ± SD	58%±7	39%±15	-	44%±16	-
Atrial fibrillation – No. (%)	-	3 (14.3%)	-	5 (16%)	-
Previous myocardial infarction – No. (%)	-	-	-	12 (37%)	-
AVB (I–III) – No. (%)	2 (9.5%)	5 (23.8%)	-	5 (16%)	-
Right bundle-brunch block – No. (%)	-	3 (14.3%)	-	2 (6%)	-
Sinus bradycardia – No. (%)	6 (28.6%)	2 (9.5%)	-	-	-
Other ECG abnormality - No. (%)	3 (14.3%)	11 (52.4%)	-	5 (16%)	-
Normal ECG - No. (%)	10 (47.6%)	-	-	3 (9%)	-
	Heart failure etiology				
Ischemic heart failure	-	-	-	14 (44%)	-
Hypertensive heart failure	-	-	-	7 (22%)	-
Dilated idiopathic heart failure	-	-	-	6 (19%)	-
Valvular heart failure	-	-	-	5 (15%)	-

Non-CARD chagasic patients: those with non-structural cardiac damage (groups A and B). CARD chagasic patients: those with structural cardiac damage (groups C and D). ACC/AHAS Classification: A: Normal electrocardiogram (ECG) and echocardiogram findings, and New York heart association functional classification (NYHA) I; B: abnormal ECG findings, normal ECHO and NYHA I; C: Abnormal ECG findings, increased heart size, decreased LVEF, and NYHA II or III; and D: same as class C, but NYHA IV. LVEF, left ventricular ejection fraction. AVB, auriculoventricular block. ECG, electrocardiogram.

### Blood sample and cell surface phenotype

Blood samples were obtained from each donor using heparin vacuntainer tubes (BD Bioscience, Franklin Lakes, NJ, USA). Blood (100 µl) was stained with two antibodies panels: a) anti-CD3 APC (clone SK7), anti-CD4 PerCP (SK3), anti-CD8 PE (RPA-T8), anti-HLA-DR PE-Cy7 (L243) and anti-CD38 FITC (HIT2); and b) anti-CD3 APC, anti-CD4 PerCP, anti-CD8 PE, anti-HLA-DR PE-Cy7 and anti-CD28 FITC (clone CD28.2). All monoclonal antibodies were purchased from BD-Pharmingen (San Diego, CA, USA). Samples were stained in darkness for 20 min at 4°C and then incubated with cell lysis buffer (BD Bioscience, San Jose, CA, USA) for 12 min at room temperature, and washed twice in PBS 0.01 M, pH 7.4 (PBS 1×). Samples were acquired in a FACS Canto II cytometer with FACSDiva software (BD Bioscience) and data analyzed with FlowJo 7.5.5 software (Tree Star, Inc. Ashland, OR, USA). At least 5×10^4^ cells were acquired in the lymphocyte population gate according to their forward scatter (FSC) versus side scatter (SCC) features; CD3+ cells were then subdivided by their expression of CD4 or CD8 markers, and the expression of CD28, CD38 and HLA-DR was assessed. Representative flow cytometry density plots showing the gating strategy are shown in Figure S1A.

### CFSE staining and proliferation assay

Isolation of PBMCs was done using ficoll-hypaque density gradient (Sigma-Aldrich, St. Louis, MO, USA). Five million PBMCs were re-suspended in PBS 1× with 0.1% FBS (Eurobio, Courtaboeuf, Les Ulis, France), and then incubated with CFSE 4 µM (Invitrogen, Eugene, Oregon, USA) for 8 min in darkness. After adding 2 ml of FBS, samples were incubated in a water bath at 37°C for 10 min and washed twice with 5% FBS in RPMI 1640. A total of 2×10^6^ PBMCs were incubated under the following conditions: medium alone, 5 µg/ml of PHA (Sigma, St. Louis, MO, USA) or *T. cruzi* trypomastigote (MHOM/CO/01/DA) lysate (10 µg/ml) during 5 days at 37°C and 5% CO_2_. For *T. cruzi* lysate preparation, MHOM/CO/01/DA trypomastigotes were harvested in PBS 1× and exposed to 4 cycles of freeze-thaw at −80°C with the addition of 10 µl/ml of proteases inhibitor cocktail (Sigma). The antigen was sonicated (20 sec×5 min) and the protein concentration was measured with a fluorometer Qubit 2.0 (Invitrogen, Carlsbad, CA, USA) [28]. Forty-eight hours after stimulation, 1 µg/ml of recombinant IL-2 (Proleukin, Novartis Vaccines & Diagnostics, Inc. Emeryville, CA, USA) was added to the cultures. Cells were then stained with anti-CD3 APC, CD4 PerCP and CD8 APC-H7 (clone SK1) during 30 min at 4°C, washed as described above and gently re-suspended. Samples were acquired in a FACS Canto II with FACSDiva software (BD Bioscience). At least 5×10^4^ cells were acquired in the lymphocyte population gate according to their forward scatter (FSC) versus side scatter (SCC) features. Dead cells were excluded by light scatter parameter (FSC-H versus FSH-A), and the above-described gating strategy was used. The proliferation analysis was done with FlowJo software on the CD3+/CD4+ or CD3+/CD8+ populations and included the determination of the division index (average number of cell divisions in the entire population), proliferative index (total number of divisions divided by the number of cells that went into division, or the average number of cell divisions in the “responder cells”) [29]; and based on these analysis we calculated the stimulation index (% of divided cells with parasite lysate/% of divided cells with medium alone). Representative flow cytometry density plots showing the gating strategy are shown in Figure S1B.

### CD3ζ intracellular staining

PBMCs were isolated as described above. After assessing cell viability by trypan blue exclusion, surface staining was done with antibodies against CD3 APC, CD4 PerCP and CD8 APC-H7 for 30 min at 4°C. Cells were then fixed with 500 µl of 2% formaldehyde for 10 min, washed and permeated with PBS 1×0.1% sodium azide, 0.1% FBS and 0.1% Tween 20 for 20 min at 4°C, followed by intracellular staining with anti-CD3ζ FITC antibody (Anti-CD247, clone 6B10.2, Biolegend, San Diego, CA, USA). Lastly, cells were washed and analyzed as described above. At least 2×10^5^ cells were recorded by flow cytometry on the CD3+ population, and CD3ζ expression (CD3ζ^bright^, CD3ζ^dim^ and CD3ζ^neg^) was determined on CD3+/CD4+ and CD3+/CD8+ gates. As control and to define the level of expression, we stained an additional sample per patient with a mouse anti-human IgG1 κ FITC isotype (BD, G18-145) instead of CD3ζ FITC with the same procedure. Representative flow cytometry density plots showing the gating strategy are shown in Figure S1C.

### *Ex vivo* influence of live parasite in PBMCs proliferation and CD3ζ expression

PBMCs from three healthy individuals were extracted as described above. A total of 3×10^6^ cells were incubated with 0.3×10^6^ *T. cruzi* trypomastigotes (MHOM/CO/01/DA) separated by a 3.0 µm pore size transwell (Corning Incorporated, NY, USA). The expression of CD3ζ chain was determined at 24 h, 48 h and 72 h of incubation as described in the previous section. Additionally, 2×10^6^ cells from each individual were also stained with CFSE and incubated with 2×10^5^ *T. cruzi* trypomastigotes (MHOM/CO/01/DA) separated with 3.0 µm pore size transwell in one of the next conditions: medium alone or 5 µg/ml of PHA. Proliferation index was measured at 5^th^ post-stimulation day.

### Flow cytometry analysis of cardiac tissue

Fresh explanted heart tissue from two patients that underwent cardiac transplant was also processed to obtain infiltrating cells. The early chronic sample came from a 24-year-old female suffering early chronic chagasic cardiomyopathy (absence of symptoms related to acute infection and undetectable parasitemia at the time of surgical procedure [13]), approximately 70 days after oral transmission. The later chronic sample came from a 46 years-old male patient with Chagas cardiomyopathy stage D, and who met the criteria for cardiac transplant. In both cases immediately after the heart extraction tissue was cut and submerged in ice-cold normal saline solution (NaCl 0.9%) and gently ground it. After decanting big pieces of tissue in a 15 ml tube, the supernatant was taken and cells were isolated using ficoll-hypaque density gradient. Cells were counted by trypan blue exclusion. One million cells were re-suspended in PBS 1×0.1% sodium azide and 0.1% FBS and stained with anti-CD3 APC, anti-CD8 APC-H7, anti-HLA-DR PE-Cy7 and anti-CD38 FITC in the early chagasic sample; in addition, the late chronic sample was also marked with anti-CD28 FITC and intracellular CD3ζ FITC. The staining procedure was carried out as described above. At least 10×10^3^ cells were recorded by flow cytometry on the CD3+ population and CD28, CD38, HLA-R and CD3ζ expression (CD3ζ^bright^, CD3ζ^dim^ and CD3ζ^neg^) was determined in CD4+ and CD8+ gates as previously shown.

### Statistical analysis

Descriptive statistics (mean, standard deviation and percentages) were used to depict the population and present the flow cytometry data. Non-parametric analyses were performed (Statistix 8.0 software) to compare groups using Kruskal Wallis test, followed by Dunn post hoc tests. Significance was considered P value<0.05.

## Results

### Chagasic patients displayed higher frequencies of T lymphocytes co-expressing CD38 and HLA-DR, and the percentage of activated CD4+ T cells is correlated with the disease stage

**Figure 1:**
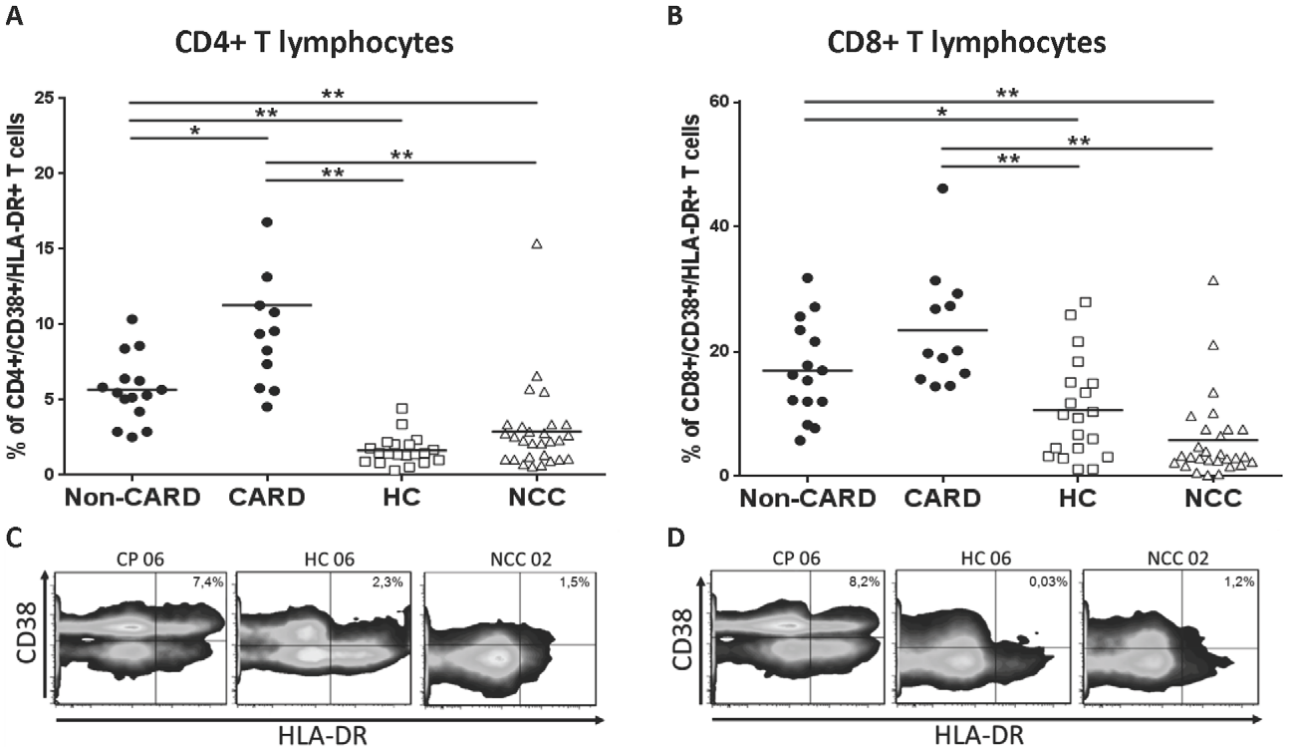
Activation markers in T cell population from chagasic patients and uninfected controls. Averaged percentages of HLA-DR+/CD38+ CD4+ (**A**) and CD8+ (**B**) T lymphocytes in chagasic patients (CP) without (stages A+B = non-CARD, black dots n = 15) and with structural cardiac damage (stages C+D = CARD, black dots n = 12), healthy controls (HC, empty squares, n = 20) and non-chagasic cardiomyopathy (NCC, empty triangles, n = 28) donors. Representative density plots of CD38 and HLA-DR expression on CD4+ (**C**) and CD8+ (**D**) T cells; the percentage of CD38+/HLA-DR+ cells is shown in each quadrant. * *P*<0.05 and ** *P*<0.001.

To determine the activation status of T cell subsets in chronic chagasic patients (CP: A+B non-CARD and C+D CARD), CD38 and HLA-DR co-expression was analyzed in their peripheral blood and compared with healthy controls (HC) and in patients with non-chagasic cardiomyopathy (NCC). The percentage of CD4+ T cells co-expressing CD38 and HLA-DR was higher in non-CARD (5.7%±2.2) and CARD CP (11.3%±7.6) than in HC (1.6±1.0, *P*<0.0001 and *P*<0.0001, respectively) and NCC (2.9±2.9, *P* = 0.0002 and *P*<0.0001) (Figure 1A). Non-CARD CP displayed lower values of CD4+/HLA-DR+/CD38+ T cells when compared with CARD CP (*P* = 0.005).

Also, non-CARD (16.9%±7.7) and CARD CP (23.4%±9.3) displayed a two-fold increase in CD8+/CD38+/HLA-DR+ T cells when compared with control groups: HC (10.6%±8.0, *P* = 0.019 and *P* = 0.0006, respectively) and NCC (5.8%±6.8, *P*<0.0001 and *P*<0.0001) donors (Figure 1B). Although no differences in CD8+/CD38+/HLA-DR+ T cells percentages were found between non-CARD and CARD CP (*P* = 0.07), it is worth to notice that the latter group showed a clear trend towards higher frequencies (Figure S2B).

### Proliferative capacity of the CD8+ T cell subset is diminished in chagasic patients

We next compared the proliferative capacity of T lymphocytes among chagasic patients and control groups by carboxyfluorescein diacetate succinimidyl ester (CFSE) staining after incubation with phytohemagglutinin (PHA) or parasite lysate. The division and proliferative indexes were similar in CD4+ T cells among all the studied groups either for cells in culture alone (division index *P* = 0.24 and proliferative index *P* = 0.93), those stimulated with *T. cruzi* lysate (*P* = 0.67 and *P* = 0.88) or with PHA (*P* = 0.12 and *P* = 0.71).

**Figure 2:**
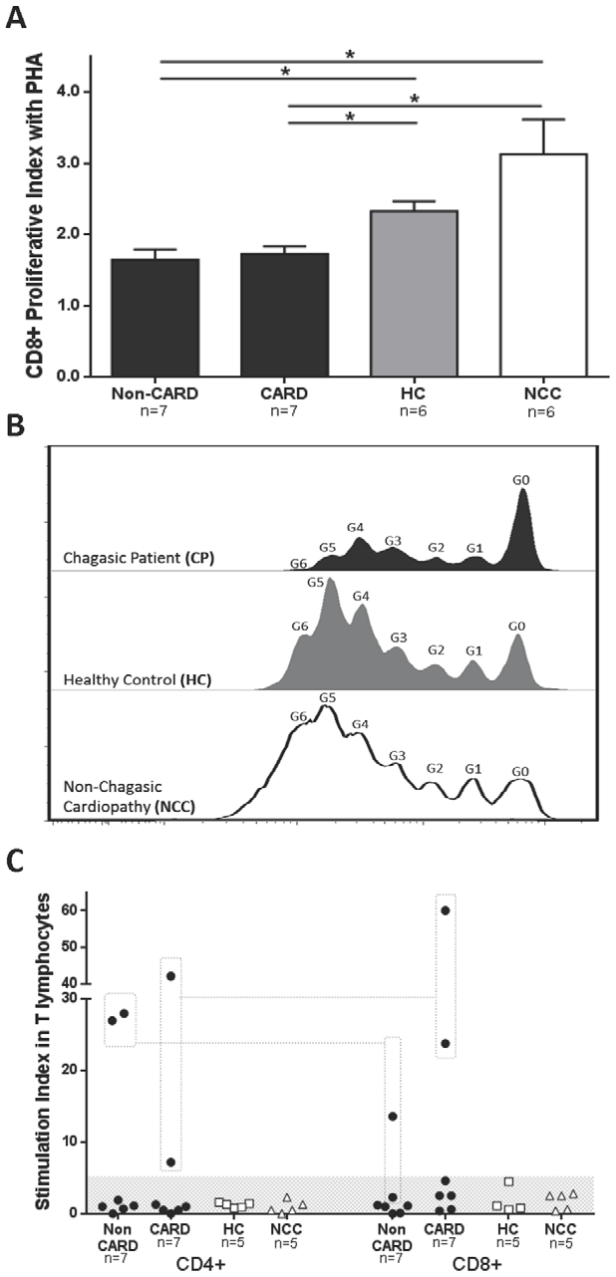
Proliferative capacity of T cell populations from chagasic patients and uninfected controls. Averaged proliferative indexes with standard deviations of CD8+ T lymphocytes (**A**) in non-CARD (black bars, n = 7) and CARD (black bars, n = 7) chagasic patients (CP), healthy controls (HC, grey bars, n = 6) and non-chagasic cardiomyopathy (NCC, empty bars, n = 6) donors after PHA stimulation. Representative histograms of CFSE fluorescence intensity for CD8+ T cells (**B**) in chagasic patients (dark histogram), healthy controls (grey histogram) and non-chagasic cardiomyopathy donors (empty histogram). The generation number (G0 through G6) is displayed. (**C**) Stimulation indexes (% of divided cells with parasite lysate/% of divided cells with medium alone) from non-CARD (black dots, n = 7) and CARD (black dots, n = 7) chagasic patients, healthy controls (HC, empty squares, n = 5) and non-chagasic cardiomyopathy (NCC, empty triangles, n = 5) donors are shown. Note that the CD4+ and CD8+ T cells from the same 2 non-CARD and 2 CARD CP respond to parasite lysate (dotted line and squares). * *P*<0.05.

In contrast, the proliferation index of CD8+ T cells after PHA stimulation was lower in non-CARD (1.6±0.4) and CARD CP (1.8±0.3) when compared with HC (2.3±0.3, *P* = 0.015 and *P* = 0.022, respectively) and NCC donors (3.1±1.1, *P* = 0.012 and *P* = 0.031) (Figure 2A). The division index of CD8+ T cells was higher in HC, when compared with all the other groups (data not shown). Division or proliferative indexes for CD8+ T cells in cultures with medium alone or with *T. cruzi* lysate did not showed any statistical difference. Representative histograms after PHA stimulation and averaged proliferative indexes for CD8+ T cell populations are shown in Figure 2A and 2B.

When stimulation indexes (% of divided cells with parasite lysate/% of divided cells with medium alone) for CD4+ and CD8+ T cells in the presence of *T. cruzi* antigen were compared, no differences were found. Nevertheless, four out of fourteen non-CARD and CARD CP displayed high stimulation indexes (28.0 and 27.0 for non-CARD and 7.2 and 42.2 for CARD) in the CD4+ T cell population. Likewise, one of non-CARD and the two same CARD CP displayed significant CD8+ stimulation indexes (13.6 non-CARD, and 23.8 and 60.0 for CARD). All the other individuals, including chagasic patients and controls, displayed stimulation indexes lower than 5 (Figure 2C).

### T lymphocytes sub-populations from chagasic patients down-regulate the expression of CD28, a co-stimulatory molecule

**Figure 3:**
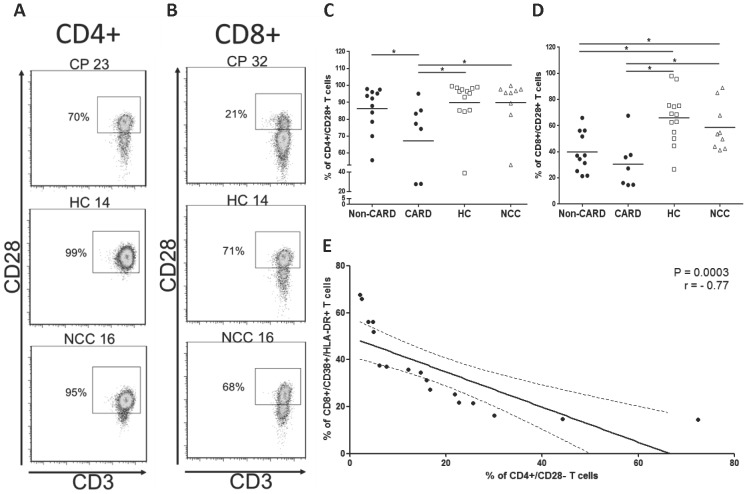
CD28 expression in T cell population from chagasic patients and uninfected controls. Representative density plots of CD28 expression on CD4+ (**A**) and CD8+ (**B**) T cells in chagasic patients (CP), healthy controls (HC) and non-chagasic cardiomyopathy (NCC) donors. The percentage of CD28+ T cells is shown next to each gate. Averaged percentage of total CD4+ (**C**) and CD8+ (**D**) T cells expressing CD28 in non-CARD (black dots, n = 11) and CARD (black dots, n = 7) chagasic patients, healthy controls (empty squares, n = 13) and non-chagasic cardiomyopathy (empty triangles, n = 9) donors. (**E**) Regression analysis between the frequency of CD4+CD28− and the fraction CD8+/CD38+/HLA-DR+ T cells in chagasic patients (non-CARD and CARD). P and *r* value are shown. * *P*<0.05 and ** *P*<0.001.

In order to elucidate the mechanism behind the proliferative dysfunction, the expression of the co-stimulatory molecule CD28 was determined on T cell sub-populations. The fraction of CD4+/CD28+ T cells was significantly lower in CARD CP (67.2±27.8) relative to non-CARD CP (86.3±13.4, *P* = 0.045), HC (89.9±16.3, *P* = 0.006) and NCC donors (89.8±14.8, *P* = 0.02) (Figure 3C). The fraction of CD8+/CD28+ T cells was also lower in non-CARD (39.9%±15.3) and CARD CP (30.9%±19.4) when compared with HC (66.1%, ±19.5 *P* = 0.0038 and *P* = 0.0043) and NCC (58.7%±18.1, *P* = 0.04 and *P* = 0.008) respectively (Figure 3D). We did not find differences when CD8+/CD28+ T lymphocytes were compared between non-CARD and CARD CP (*P* = 0.28). Representative flow cytometry dot plots and percentages of CD28+ T cell are shown in Figure 3A and 3B.

Interestingly, we found a negative correlation (*P* = 0.0003, *r* = −0.77) among the percentage of CD4+/CD28− and the fraction of activated CD8+ T cells (CD38+/HLA-DR+) in the entire cohort of CP (both non-CARD and CARD) (Figure 3E). No other correlations were found among the studied populations.

### CD3ζ intracellular expression is decreased in the CD4+ and CD8+ T cell subsets from chagasic patients

**Figure 4:**
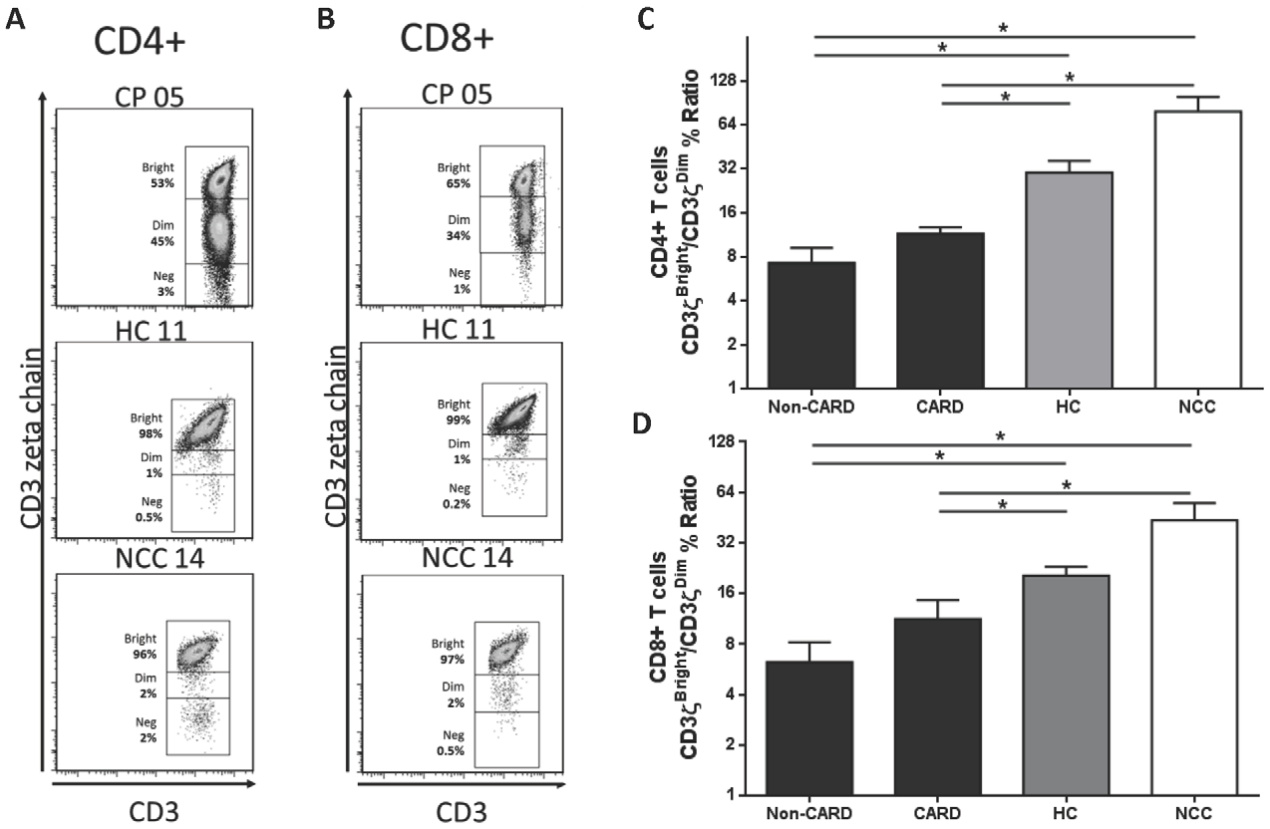
CD3ζ expression in T cell population from chagasic patients and uninfected controls. Representative density plots of expression of CD3ζ expression on CD4+ (**A**) and CD8+ (**B**) T lymphocytes of chagasic patients (CP), healthy controls (HC) and non-chagasic cardiomyopathy (NCC) donors; the percentage of CD3ζ^bright^, CD3ζ^dim^ and CD3ζ^neg^ T cells is shown next to each gate. The averaged ‘percentage of CD3ζ^bright^/percentage of CD3ζ^dim^’ ratio with standard deviation of CD4+ (**C**) and CD8+ (**D**) T lymphocytes of non-CARD (black bars, n = 6) and CARD (black bars, n = 6) chagasic patients, healthy controls (HC, grey bars, n = 6) and non-chagasic cardiomyopathy (NCC, empty bars, n = 6) donors is displayed. * *P*<0.05.

We next assessed the intracellular expression of CD3ζ in T lymphocytes sub-populations. The CD3ζ^bright^/CD3ζ^dim^ % ratio for CD4+ T cells was lower in non-CARD (7.3±4.8) and CARD CP (11.5±3.0) when compared with HC (30.0±15.0, *P* = 0.005 and *P* = 0.01) and NCC (78.7±49.6, *P* = 0.03 and *P* = 0.04) (Figure 4C). We did not find differences when CD4+ CD3ζ^bright^/CD3ζ^dim^ % ratio was compared between CARD and non-CARD (*P* = 0.11). Consistently, the CD3ζ^bright^/CD3ζ^dim^ % ratio for CD8+ T cells was lower in non-CARD (6.2±4.8) and CARD CP (11.2±8.2) when compared with HC (20.4±6.5, *P* = 0.004 and *P* = 0.047) and NCC (43.6±28.3, *P* = 0.030 and *P* = 0.045) (Figure 4D). There was no difference when CARD and non-CARD were compared (*P* = 0.23). Representative flow cytometry dot plots of CD3ζ expression on CD4+ and CD8+ T cell populations are shown in Figure 4A and 4B, respectively.

### PBMCs indirect culture with live parasite causes CD3ζ down-regulation and diminished proliferative response to PHA on T cells

**Figure 5:**
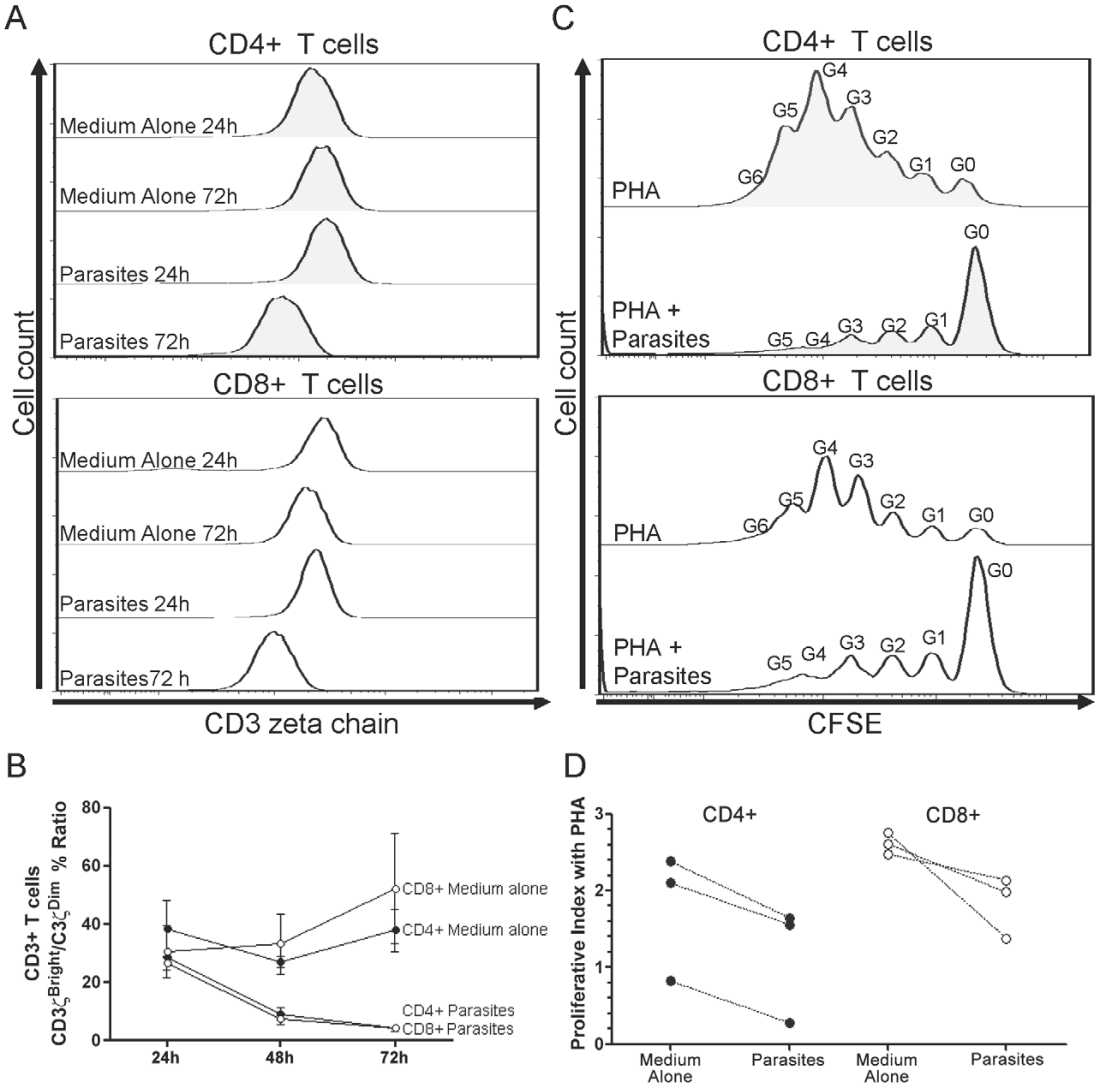
*Ex vivo* influence of live parasites on PBMC CD3ζ expression and proliferation in healthy donors. Averaged “CD3ζ^bright^/CD3ζ^dim^ %” ratio of CD4+ (black dots) and CD8+ (empty dots) (**B**) T cells incubated with medium alone or transwell-separated *T. cruzi* trypomastigotes at 24 h, 48 h and 72 h. Averaged proliferative index after PHA stimulation of CD4+ (black dots) and CD8+ (empty dots) (**D**) T cells incubated with medium alone or transwell-separated *T. cruzi* trypomastigotes. Representative histograms of MFI for CD3ζ at 24 h and 72 h (**A**) and CFSE at 5^th^ post-stimulation day with PHA (**C**) incubated with medium alone or *T. cruzi* trypomastigotes. The generation number (G0 through G6) is displayed.

PBMCs from healthy controls were incubated with *T. cruzi* trypomastigotes separated by transwells to avoid the infection of macrophages and dendritic cells and to allow circulation on the media of parasite-secreted antigens. The CD3ζ^bright^/CD3ζ^dim^ % ratio for CD4+ T lymphocytes decreased gradually in those cells that were cultivated with parasites (24 h = 28.6±0.7, 48 h = 8.9±3.1 and 72 h 4.0±0.8), and displayed lower values than those incubated with medium alone (24 h = 38.2±13.9, 48 h = 33.10.2±5.4 and 72 h = 37.8±10.3). Similar results were found for CD8+ population, the ratios of CD3ζ^bright^/CD3ζ^dim^ were lower in the cells cultivated with parasites (24 h = 26.6±3.3, 48 h = 7.3±2.6 and 72 h = 4.2±1.6) than in those with medium alone (24 h = 30.4±12.9, 48 h = 33.1±14.8 and 72 h = 52.1±26.1). Representative histograms and averaged CD3ζ^bright^/CD3ζ^dim^ % ratios are presented in Figure 5A and 5B.

PBMCs from uninfected donors displayed lower proliferative indexes in both CD4+ and CD8+ T cells when incubated with parasites and PHA (CD4+ 1.2±0.6, CD8+ 1.83±0.3) than those cells incubated just with PHA (CD4+ 1.8±0.7, CD8+ 2.6±0.1) (Figure 5C and 5D).

### Heart infiltrating T cells from chagasic patients also express activation markers and down regulates the expression of CD28 and CD3ζ

**Figure 6:**
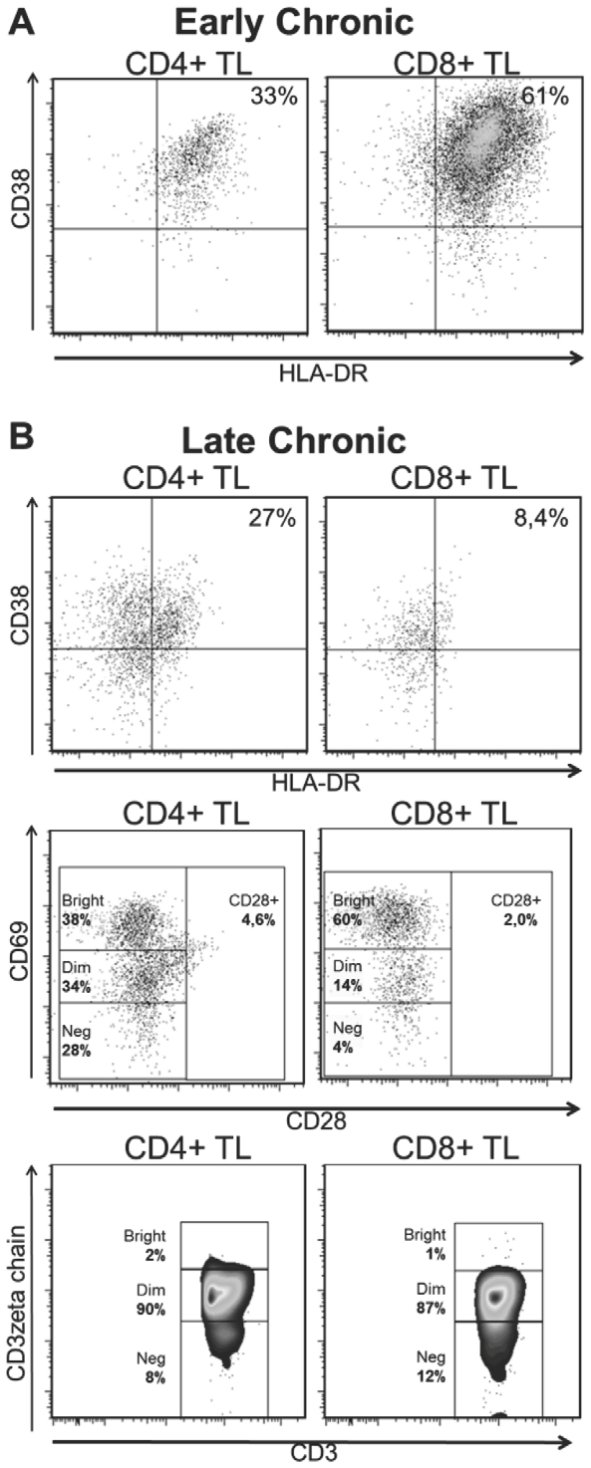
Phenotypic analysis of heart infiltrating T cells in early and late chronic chagasic cardiomyopathy. Dot and density plots of expression of CD38 and HLA-DR, CD69, CD28, and intracellular CD3ζ chain in CD4+ and CD8+ heart infiltrating T lymphocytes from an early chronic (**A**, n = 1) and late chronic (**B**, n = 1) chagasic patient.

In order to characterize the activation status and expression of co-stimulatory molecules on infiltrating T lymphocytes in chagasic patients, mononuclear cells extracted from explanted hearts of two infected individuals were phenotypically analyzed. In the early chronic chagasic patient, 80% of the total CD3+ lymphocytes infiltrating the heart were CD8+, 13% were CD4+, and 1% were CD4+/CD8+ double positive T cells. Thirty percent of the CD4+ and 61% of the CD8+ T cells were HLA-DR+/CD38+. In the late chronic stage, 42.2% and 37.9% of the total CD3+ T cells were CD4+ and CD8+, respectively, and 5.7% were CD4+/CD8+ double positive. Among CD4+ T cells, 27% were CD38+/HLA-DR+, 4.6% were CD4+/CD28+, 38% were CD69^bright^ and the CD3ζ^bright^/CD3ζ^dim^ % ratio was 0.02. In the CD8+ T lymphocytes, 7.4% co-expressed CD38 and HLA-DR, 2.0% were CD28+, 60% were CD69^bright^ and the CD3ζ^bright^/CD3ζ^dim^ % ratio was 0.01. Flow cytometry density and dot plots are shown in Figure 6A and 6B.

## Discussion

The immune mechanisms implicated in chronic Chagas disease's pathogenesis are not completely understood, but experimental evidence suggests that parasite persistence can promote an overactive and dysfunctional cellular immune response [12], [13]. This study confirms previous findings by other groups [20]–[23] where T cells from chronic chagasic patients are over-activated; and furthermore it shows that the percentage of CD4+/HLA-DR+/CD38+, and probably CD8+/HLA-DR+/CD38+ T cells, is correlated with disease severity. One previous study showed that adults with chronic Chagas disease displayed higher percentages of CD4+/HLA-DR+ T cells when compared with controls [20], and only those with severe cardiac compromise had higher CD8+/HLA-DR+ [20], [24]; nonetheless, others studies have found contradictory results regarding the expression of either HLA-DR or CD38 on T cell populations from chronic chagasic patients [21]–[23]. Here, we report that both activation markers are consistently and simultaneously over-expressed in the two main peripheral T cell populations of all chagasic patients.

In other infectious models, the co-expression of CD38 and HLA-DR is used to identify the recently activated T cells [2], and some of these works indicate that CD38+/HLA-DR+ T lymphocytes can be persistently expanded as a result of chronic antigen exposure [6], [7]. For example, in HIV+ individuals the percentage of these T cells correlated with the immune dysfunction and disease progression [9]; and furthermore they failed to proliferate in response to mitogen stimulation [30]. In our study, we simultaneously found an over-activated status and proliferative dysfunction on T lymphocytes, based on previous findings in HIV+ patients, these two phenomena appear to be related [30].

Abnormalities in T lymphocyte proliferation are described in *T. cruzi*-infected mice [31] and in human PBMCs exposed *in vitro* to *T. cruzi* antigens [32], [33]; and have been related with decreased expression of IL-2 and IL-2R, in addition to diminished cyclin D2 phosphorylation [34]. Although, the mechanisms of proliferative dysfunction on CD8+ T cells in Chagas disease need further research, previous studies suggest that they could be related to a decreased expression of co-stimulatory molecules, γ common receptor cytokine starvation or expression of inhibitory receptors as PD-1 [11], [17].

Simultaneously with the increased activation status of peripheral T cells, the expression of CD3ζ and CD28 was significantly diminished in chagasic patients. The down-regulation of CD28 is consistent with the over-activated status of T lymphocytes. Indeed, it is known that prolonged antigenic stimulation of T cells promotes down-regulation of CD28 [35]. In other infectious models, the patency of the CD28/B7.1(CD80) co-stimulatory pathway has proven to be essential to prevent the induction of clonal anergy and to overcome the IL-10 induced T cell proliferative inhibition [25]. *In vitro* studies showed that CD28− T cells have diminished proliferative potential associated with decreased length of telomeres and replicative senescence [36]. In murine models, the absence of CD28 on T lymphocytes is associated with a high susceptibility to *S. thyphimurium* [37], *L. monocytogenes* [38] and *T. gondii* [39]. Consistently, CD28 knockout mice infected with *T. cruzi* showed reduced IFN-y and IL-2 production, decreased T cell proliferation and consequently increased parasite burden [40], [41]. Furthermore, in human Chagas disease higher percentages of CD28− T cells have been associated with increased IL-10 production [42], [43]. In our study CD4+/CD28− T cells were negatively correlated with the amount of activated CD8+ T cells. Preserved functions of the CD4+ T cells are required for the secondary expansion and the maintenance of memory CD8+ T lymphocytes [44]; therefore this aforementioned negative association suggests that the defects of CD28 expression on CD4+ T cells might affect the activation, and probably proliferation, of the CD8+ T cells. Similar phenomena have been described in HIV+ individuals where the percentage of CD4+/CD28−, but not of CD8+/CD28−, T cells is associated with disease progression and patients overall survival [45], [46].

Moreover and consistent with our results, previous studies have shown that down-regulation of CD28 and CD3ζ are associated events that can occur simultaneously during acute or chronic inflammatory diseases including HIV [47] and EBV infection [26]. Down-regulation of CD3ζ has also been described in inflammatory or immune mediated diseases including rheumatoid arthritis [48], systemic lupus erythematosus [49] and leprous [50]. Interestingly, in these diseases the down-regulation CD3ζ-chain was associated with reduced IL-2 and IL-2R expression [26], [51], decreased proliferative capacity [52] and high IL-10 secretion [25], [53] on T cells.

The study of the heart-infiltrating T cells from two chagasic patients showed that on average, 30% of the CD4+ and 60% of the CD8+ T cells expressed early (CD69) or late activation markers (CD38 and HLA-DR). There was an interesting shift from a CD8+/HLA-DR+/CD38+ dominant pattern in the ‘early stage’ to a CD8+/CD69+ in ‘late chronic stage’. Also, the frequency of CD4+/CD8+ double-positive (DP) T cells increased in the late chronic stage compared with the early chronic subject. The role of these cell populations in chronic tissue damage needs further investigation, however it is known that CD38+/HLA-DR+, CD8+/CD69+ and DP T cells can produce perforin and IFN-γ in chagasic patients [2], [12], [54]; and previous studies have indirectly implicated DP T cells in tissue damage in Chagas disease [12] and other infectious models [55]. Nevertheless, these results remain descriptive based on the limited availability of samples, and certainly this topic needs further research.

Our results suggest that, at least to some extend, the proliferative dysfunction and CD3ζ/CD28 down-regulation might be related to parasite persistence during chronic Chagas disease; and furthermore, can be reproduced *in vitro* when PBMC from healthy individuals are indirectly exposed to live parasites. Certainly, to determine if these *in vitro* conditions somehow mimic the T cell environment in human Chagas diseases is challenging; nonetheless, recent evidence suggests that the parasitemia in the chronic stages of the disease is not static, and in fact can occasionally reach high values [56]–[58]. Moreover, our results showed that CARD CP had lower CD8+ proliferative response to PHA, low level of T cells expressing CD28+ cells, lower expression CD3ζ (CD3ζ ^bright^/CD3ζ ^dim^ Ratio) and still higher fractions of activated T cells than individuals with non-chagasic cardiomyopathy; suggesting that parasite persistence, more than the systemic changes derived from heart failure, are associated with these cellular immune abnormalities.

Overall, this study showed that the percentages of T cells expressing activation markers in chagasic patients was augmented, while the expression of CD28 and CD3ζ chain as well as the CD8+ proliferative capacity were diminished. In addition, the percentage of CD4+/HLA-DR+/CD38+ and CD4+/CD28− correlated with disease stage. Based on previous evidence from other chronic infectious models, it seems plausible that all these features from the cellular immune response are closely related, and could be a manifestation of the same biological process. Furthermore, this study reports some cellular immune abnormalities almost certainly caused by *T. cruzi* antigen persistence, and contribute novel insights to the pathogenesis of chronic Chagas disease.

## Supporting Information

Figure S1 **Gating strategy for 6-color flow cytometry.** (**A**) Representative donor peripheral blood analysis, whereby gated populations (from left to right) are indicated, defining viable CD3+CD4+ and CD3+CD8+ T cells; the expression of CD28 and co-expression of CD38/HLA-DR was determined on these populations. (**B**) Representative donor PBMC analysis done by FlowJo, whereby the CFSE fluorescence was determined in the gated populations: viable CD3+CD4+ and CD3+CD8+ T cells. Each peak was considered as a generation. (**C**) Representative donor PBMC analysis, whereby the CD3ζ expression was determined in the gated of viable CD3+CD4+ and CD3+CD8+ T cells. The cut-off expression for CD3ζ was based on the fluorescence of an anti-human IgG1 isotype, as indicated. Viable CD3+CD4+ and CD8+ T cells are differentiated into CD3ζ^bright^, CD3ζ^dim^ and CD3ζ^neg^ cells.
(TIFF)

Figure S2 **Phenotypic characterization and proliferative capacity of T cell populations from chagasic patients according to disease stage.** The averaged expression plus standard deviation of CD38/HLA-DR and CD28 in CD4+ (**A** and **E**) and CD8+ (**B** and **F**) T cells according to disease stage (**A** trough **D**) is displayed. Additionally, the averaged proliferative index and the “percentage of CD3ζ^bright^/percentage of CD3ζ^dim^” ratio plus standard deviation of CD4+ (**C** and **G**) and CD8+ (**D** and **H**) T cells according to disease stage are shown. * *P*<0.05.
(TIFF)
