# Supplementary figures and images for: T Lymphocytes from Chagasic Patients Are Activated but Lack Proliferative Capacity and Down-Regulate CD28 and CD3ζ
Source: PLoS Negl Trop Dis. 2013 Jan 31;7(1):e2038. doi: 10.1371/journal.pntd.0002038 (PMC3561132; doi:10.1371/journal.pntd.0002038)

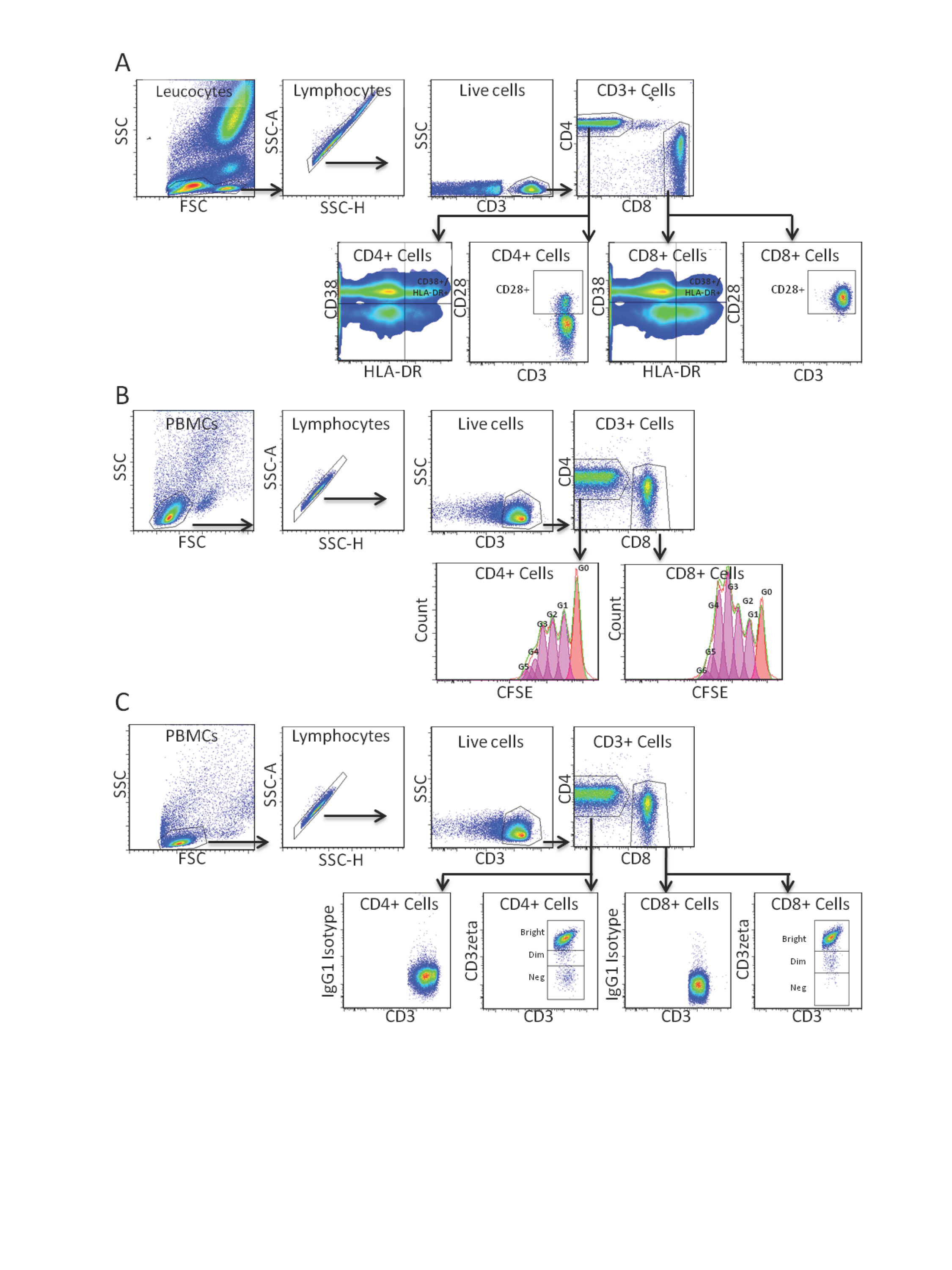

Supplement: Figure S1 — Gating strategy for 6-color flow cytometry. (A) Representative donor peripheral blood analysis, whereby gated populations (from left to right) are indicated, defining viable CD3+CD4+ and CD3+CD8+ T cells; the expression of CD28 and co-expression of CD38/HLA-DR was determined on these populations. (B) Representative donor PBMC analysis done by FlowJo, whereby the CFSE fluorescence was determined in the gated populations: viable CD3+CD4+ and CD3+CD8+ T cells. Each peak was considered as a generation. (C) Representative donor PBMC analysis, whereby the CD3ζ expression was determined in the gated of viable CD3+CD4+ and CD3+CD8+ T cells. The cut-off expression for CD3ζ was based on the fluorescence of an anti-human IgG1 isotype, as indicated. Viable CD3+CD4+ and CD8+ T cells are differentiated into CD3ζbright, CD3ζdim and CD3ζneg cells. (TIFF) [file pntd.0002038.s001.tiff]

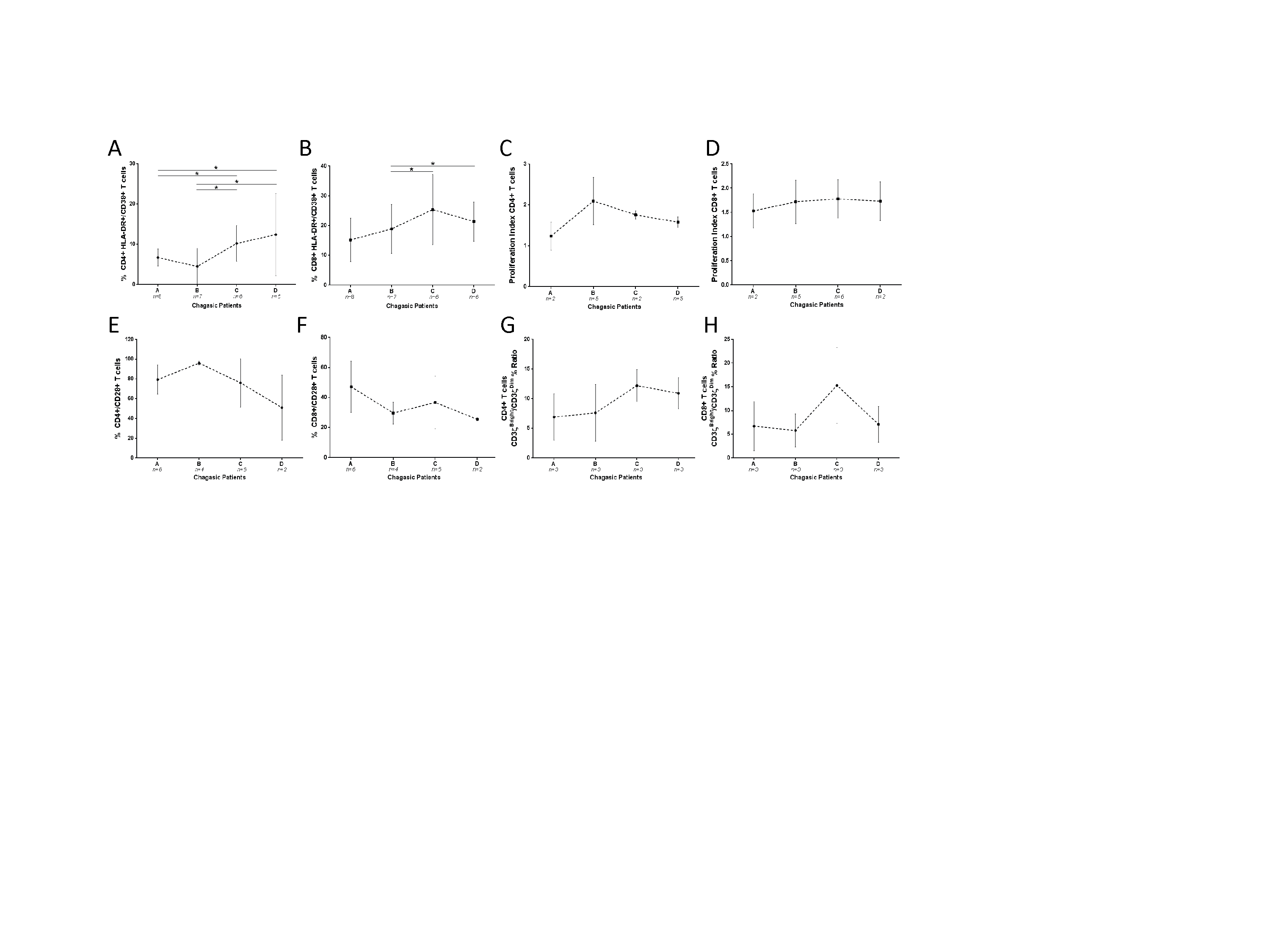

Supplement: Figure S2 — Phenotypic characterization and proliferative capacity of T cell populations from chagasic patients according to disease stage. The averaged expression plus standard deviation of CD38/HLA-DR and CD28 in CD4+ (A and E) and CD8+ (B and F) T cells according to disease stage (A trough D) is displayed. Additionally, the averaged proliferative index and the “percentage of CD3ζbright/percentage of CD3ζdim” ratio plus standard deviation of CD4+ (C and G) and CD8+ (D and H) T cells according to disease stage are shown. * P<0.05. (TIFF) [file pntd.0002038.s002.tiff]
